# NGLY1 deficiency: Novel patient, review of the literature and diagnostic algorithm

**DOI:** 10.1002/jmd2.12086

**Published:** 2020-01-30

**Authors:** Patryk Lipiński, Anna Bogdańska, Agnieszka Różdżyńska‐Świątkowska, Aldona Wierzbicka‐Rucińska, Anna Tylki‐Szymańska

**Affiliations:** ^1^ Department of Pediatrics, Nutrition and Metabolic Diseases The Children's Memorial Health Institute Warsaw Poland; ^2^ Department of Biochemistry, Radioimmunology and Experimental Medicine The Children's Memorial Health Institute Warsaw Poland; ^3^ Anthropology Laboratory The Children's Memorial Health Institute Warsaw Poland

**Keywords:** alacrimia/hypolacrimia, congenital disorder of deglycosylation, global developmental delay, hyperkinetic movement disorder, N‐glycanase 1 deficiency

## Abstract

**Objectives:**

Together with the lysosomal storage diseases, NGLY1 deficiency is a congenital disorder of deglycosylation (NGLY1‐CDDG). Since the first report in 2012, 26 patients have been described. All but one were diagnosed by exome or genome sequencing; the remaining one was identified by finding an increased concentration of an urinary marker.

The aim of this study was to describe the clinical, biochemical, and molecular features of the first Polish patient diagnosed with NGLY1‐CDDG, to provide an overview of the literature and to propose a diagnostic algorithm.

**Results:**

A Polish patient presented with global developmental delay, hyperkinetic movement disorder, stagnation of head growth, hypolacrimia, elevated serum transaminases, and hypolipidemia in infancy. Whole exome sequencing revealed two heterozygous nonsense variants in the *NGLY1* gene (a novel and an unreported). Literature review revealed global developmental disability in all reported patients, and hyperkinetic movements as well as alacrima/hypolacrima in nearly all.

**Conclusions:**

NGLY1‐CDDG should be considered in patients with developmental disability associated with a hyperkinetic movement disorder and alacrimia/hypolacrima. Absence of the latter two symptoms does not rule out this diagnosis.

**Abbreviations**
ALT: alanine aminotransferase
ApoA1: apolipoprotein A1
ApoB: apolipoprotein B
AST: aspartate aminotransferase
DBS: dried blood spot
DI‐HRMS: direct‐infusion high‐resolution mass spectrometry
HDL‐C: high density lipoprotein‐cholesterol
LDL‐C: low density lipoprotein‐cholesterol
MALDI‐TOF: matrix assisted laser desorption time of flight
NGLY1: N‐glycanase 1
NGLY1‐CDDG: NGLY1 congenital disorder of deglycosylation
TC: total cholesterol
TG: total triglycerides
ULN: upper limit of normal range
VLDL‐C: very low density lipoprotein‐cholesterol
WES: whole exome sequencing

## 1 INTRODUCTION

N‐glycanase 1 (EC 3.5.1.52), also known as peptide‐N(4)‐(N‐acetyl‐beta‐glucosaminyl) asparagine amidase or cytosolic PNGase, is a de‐*N*‐glycosylating enzyme, encoded by the *NGLY1* gene, responsible for the removal of *N*‐linked or asparagine‐linked glycans (*N*‐glycans) from glycoproteins.^1^, ^2^ Together with the lysosomal storage disorders, N‐glycanase 1 deficiency (NGLY1 deficiency, OMIM 615273) is a congenital disorder of deglycosylation (NGLY1‐CDDG).^1^, ^2^ Since the first report in 2012 by Need et al, 26 patients with a confirmed molecular diagnosis have been described,^3^, ^4^, ^5^, ^6^, ^7^, ^8^, ^9^, ^10^, ^11^, ^12^ and 29 patients have been included in a registry initiated by the Grace Science Foundation in 2017.^5^

The pathogenesis of N‐glycanase 1 deficiency remains unknown, but a cytosolic accumulation of misfolded glycoproteins as well as a dysregulation of the endoplasmic reticulum‐associated degradation (ERAD) pathway were described.^3^ The clinical features of NGLY1‐CDDG include a significant developmental disability, abnormal involuntary movements, alacrimia or poor tear production, hypotonia, microcephaly, as well as elevated serum transaminases.^3^, ^4^, ^5^, ^6^, ^7^, ^8^, ^9^, ^10^, ^11^, ^12^ All patients except one have been diagnosed by exome or genome sequencing; the nonsense variant c.1201A>T (p.R401X) in the *NGLY1* gene is the most common one.^3^, ^4^, ^5^, ^6^, ^7^, ^8^, ^9^, ^10^, ^11^, ^12^ Recently, two potential biomarkers have been described—a urinary biomarker identified by oligosaccharide analysis using matrix assisted laser desorption—time of flight (MALDI‐TOF) mass spectrometry and aspartylglucosamine identified in dried blood spots (DBS) with direct‐infusion high‐resolution mass spectrometry (DI‐HRMS).^10^, ^12^ One NGLY1‐CDDG patient was identified by finding an increased concentration of this urinary marker.

The aim of this study was to describe the clinical, biochemical, and molecular features of the first patient diagnosed with NGLY1‐CDDG in Poland, to review the literature and to propose a diagnostic algorithm.

## 2 METHODS

The presentation at the time of diagnosis and detailed follow‐up were described. Details on anthropological assessment, analysis of lipid serum profile, transient elastography with FibroScan and molecular analysis are available as Supporting Information. An informed and written consent was obtained from the parents of the patient. Ethical approval of the study protocol was obtained from the Children's Memorial Health Institute Bioethical Committee, Warsaw, Poland.

A literature search through MEDLINE and PubMed up to September 2019 was performed using the search terms “NGLY1 deficiency” and “NGLY1‐congenital disorder of deglycosylation.”

## 3 RESULTS

### 3.1 Patient presentation

**Figure 1:**
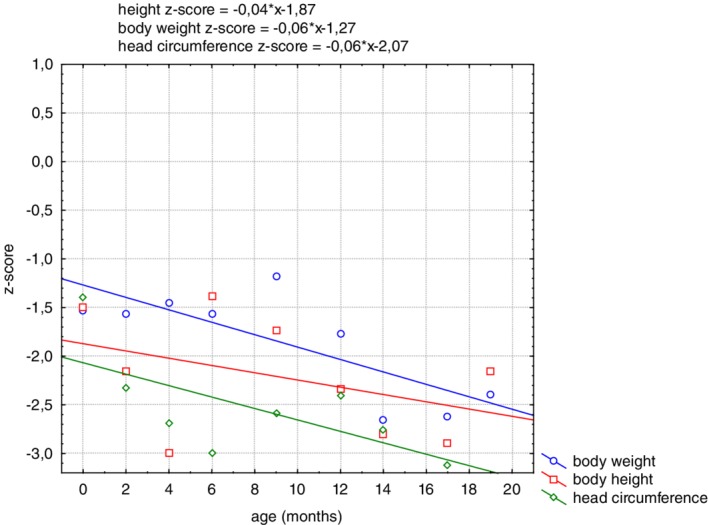
The straight line regression of standardized body height, weight, and head circumference against the adopted reference system

**Figure 2:**
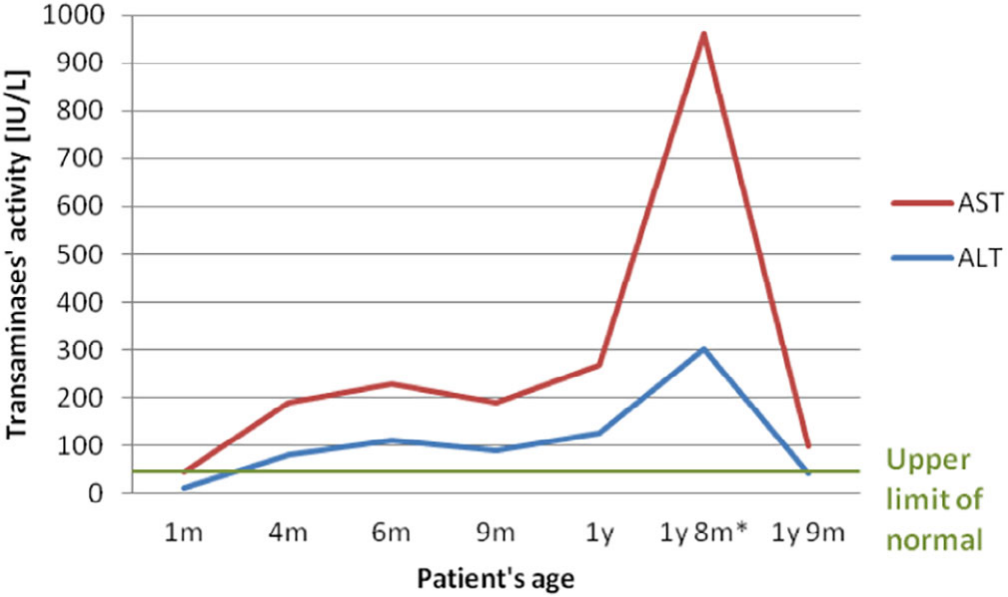
Serum transaminases in NGLY1‐CDDG patient. *Elevation during infection. ALT, alanine aminotransferase; AST, aspartate aminotransferase

The patient was the third child of nonconsanguineous healthy Polish parents born at 37 weeks of gestation via caesarean section due to fetal distress on cardiotocography. Birthweight was 2580 g, length 49 cm, and head circumference 33 cm. Poor weight gain was observed since birth (Figure 1). From the age of 2 months head growth stopped. Elevated serum transaminases were first noted at the age of 4 months (Figure 2). Liver volume was normal. Infectious causes of serum transaminases elevation were not found. At the age of 10 months hypolipidemia was found (Table 1). Coagulation studies showed normal results. Developmental disability (including motor and speech delay), axial hypotonia, and hyperkinetic movement disorder were observed at 12 months of age. Brain MRI imaging was normal. Ophthalmic examination was normal except for hypolacrimia on Schirmer II testing.^14^ Electrocardiographic and echocardiographic results were normal. Audiologic assessment, including otoacoustic emissions and auditory brainstem response were normal. Inborn errors of metabolism were ruled out by urine organic analysis with GC/MS profiling, acylcarnitine profile by tandem mass spectrometry analysis (MS/MS) of DBS, serum transferrin IEF, blood ammonia and lactate, serum amino acids, and urinary glycosaminoglycans testing.

**Table 1:** Lipid serum profile in NGLY1‐CDDG patient consistent with hypolipidemia/hypolipoproteinemia

Parameter and reference ranges	Results in patient		
	10 m	12 m	18 m
LDL‐C [mg/dL] 96‐130	24	34	55
VLDL‐C [mg/dL] 5‐20	n.a.	6	20
HDL‐C [mg/dL] 40‐80	50	52	24
TC [mg/dL] 140‐190	85	92	99
TG [mg/dL] 50‐150	53	38	115
Apo‐AI [g/L] 1.09‐1.84	1.63	2.4	1.4
Apo‐B [g/L] 0.63‐1.88	0.29	0.6	0.3
Lp (a) [mg/dL] 0‐30	2.0	7.0	8.0

Abbreviations: m, months; n.a., not analyzed.

Whole exome sequencing (WES) showed two nonsense variants in the *NGLY1* gene (NM_018297.3): c.250G>T (p.E84X), a novel variant, and c.1201A>T (p.R401X), a known variant. The parents were carriers of these variants. The c.250G>T (p.E84X) variant was predicted in silico to be likely pathogenic.^15^

During the follow‐up elevated serum transaminases (Figure 2) and hypolipidemia (Table 1) persisted. At the age of 20 months the patient was hospitalized due to bacterial pneumonia and an episode of liver disease with hepatomegaly and a more than 10 times of ULN elevation of serum transaminases (AST > ALT) developed. No coagulopathy or hypoalbuminemia were observed. Serum ALT and liver volume normalized over the next month. Transient elastography with FibroScan revealed normal liver stiffness (*E* = 4.1 kPa; liver stiffness normal results <7 kPa).

There was a decline of the growth velocity particularly of the head growth and of the weight (Figure 1 and Table S1).

### 3.2 Literature review

Up to now, 26 patients with pathogenic *NGLY1* variants have been described in the literature. The mean age at diagnosis was 9 years of age (range 9 months ‐ 26 years). Clinical, biochemical, and molecular features of the reported 26 patients and of our patient are summarized in Tables 2 and 3. To be noted that a detailed clinical presentation was not available in almost all of the reported cases.

**Table 2:** Features of reported NGLY1‐CDDG patients

Age at diagnosis	1 y	3 y	5 y	20 y	4 y	2 y	5 y	9 m	3 y	16 y	14 y
Alive	+	n.k.	+	+	+	+	+	Died at 9.5 m	Died at 5 y	+	+
Global developmental disability	+	+	+	+	+	+	+	+	+	+	+
Movement disorder	+	+	+	+	+	+	+	+	+	+	+
Hypolacrima or alacrima	+	+	+	+	+	+	+	−	+	+	+
Elevated serum transaminases	+	+	+	+	+	+	+	n.k.	+	−	+
Biochemical diagnosis based on urinary or DBS marker	−	−	−	−	−	−	−	−	−	−	−
Genotype	c.1201A>T (p.R401X)/c.250G>T (p.Glu84X)	c.1891delC (p.Q631S)/c.1201A>T (p.R401X)	c.C1891del (p.Q631fs)/c.1201A>T (p.R410X)	c.1370dupG (p.R458fs)/c.1370dupG (p.R458fs)	c.1205_1207del (p.402_403del)/c.1570C>T (p.R524X)	c.1201A>T (p.R401X)/c.1201A>T (p.R401X)	c.1201A>T (p.R401X)/c.1201A>T (p.R401X)	c.1201A>T (p.R401X)/c.1201A>T (p.R401X)	c.1201A>T (p.R401X)/c.1201A>T (p.R401X)	c.1201A>T (p.R401X)/c.1201A>T (p.R401X)	c.347C>G (p.S116X)/c.881+5G (p.IVS5+5G>T)
References	Present patient	3	6	6	6	6	6	6	6	6	7

Age at diagnosis	9 y	16 y	3 y	5 y	6 y	8 y	10 y	17 y	7 y	n.d.
Alive	+	Died at 16 y	+	+	+	+	+	+	+	+
Global developmental delay	+	+	+	+	+	+	+	+	+	+
Movement disorder	+	+	+	+	+	+	+	+	n.k.	n.k.
Hypolacrima or alacrima	+	+	+	+	+	+	+	+	+	n.k.
Elevated serum transaminases	−	+	+	+	+	+	+	+	−	n.k.
Biochemical diagnosis based on urinary or DBS marker	−	−	−	−	−	−	−	−	+	−
Genotype	c.1533_1536del (p.A511X)/c.1533_1536del (p.A511X)	c.1533_1536del (p.A511LX)/c.1533_1536del (p.A511X)	c.953T>C (p.L318P)/c.1169G>C (p.R390P)	c.931G>A (p.E311K)/c.730T>C (p.W244R)	c.1604G>A (p.W535X)/c.1910delT (p.L637X)	c.622C>T (p.Q208X)/c.930C>T (p.G310G)	c.622C>T (p.Q208X)/c.930C>T (p.G310G)	c.1201A>T (p.R401X)/c.1201A>T (p.R401X)	c.1891delC (p.G631Sfs)/c.1891delC (p.G631Sfs)	c.1837del (p.Gln613fs)/c.1837del (p.Gln613fs)—not identified in ClinVar
References	8	8	9	9	9	9	9	9	10	11

Age at diagnosis	18 y	26 y	11 y	6 y	15 y	5 y
Alive	+	+	+	+	+	+
Global developmental delay	+	+	+	+	+	+
Movement disorder	+	+	−	−	+	+
Hypolacrima or alacrima	−	+	+	+	−	+
Elevated serum transaminases	−	−	+	+	+	+
Biochemical diagnosis based on urinary or DBS marker	+	−	+	+	+	−
Genotype	c.247‐2A>G (p.?)/c.247‐2A>G (p.?)	c.247‐2A>G (p.?)/c.247‐2A>G (p.?)	c.1756C>T (p.R586X)/c.1756C>T (p.R586X)	c.1756C>T (p.R586X)/c.1756C>T (p.R586X)	c.1853T>G (p.L618X)/c.1612_1614dupGGTA (p.T539Gfs)	c.1405C>T (p.Arg469X)/c.1405C>T (p.Arg469X)
References	12	12	12	12	12	13

Abbreviation: n.k., not known.

**Table 3:** Summary of features in reported NGLY1‐CDDG patients

Feature	N	%
c.1201A>T (p.R401X) variant on at least one allele	15/54	28
Global developmental disability	27/27	100
Hyperkinetic movement disorder	23/25	92
Hypolacrima or alacrima	24/26	92
Elevated serum transaminases	20/25	80
Biochemical diagnosis based on urinary or DBS marker	4/27	15

Abbreviation: DBS, dried blood spot.

## 4 DISCUSSION

Our patient shows all the clinical features of NGLY1 deficiency as summarized in Tables 2 and 3. All or nearly all reported patients showed psychomotor disability, a hyperkinetic movement disorder, and alacrimia/hypolacrima. In the great majority of patients, serum transaminases were increased. It is not clear whether the hypolipidemia is part of the syndrome. It is not reported in the other patients, and serum lipids have not been measured in the parents.

As to the reported variants, 15 patients were homozygous for a variant and 12 patients showed a combined heterozygosity. So far, the nonsense c.1201A>T (p.R401X) variant has been reported as the most common deleterious allele identified and it was supposed to have originated in Europe.^6^, ^9^ The molecular overview of all reported patients (Tables 2 and 3) showed that this variant comprised approximately one third of all identified alleles. Some authors reported that its presence on at least one allele could be associated with a more severe clinical phenotype. Three out of all described patients have already died. One out of eight patients reported by Enns et al died at 5 years of age following a viral illness complicated by prolonged seizures; the second one died at 9.5 months of age in the sleep and the cause of death remains unknown.^6^ Both patients were homozygotes for the nonsense c.1201A>T (p.R401X) variant, which may confirm the correlation between a more severe clinical phenotype and the presence of c.1201A>T (p.R401X) variant on at least one allele. The patient described by Caglayan et al died at the age of 16 years due to respiratory failure secondary to recurrent respiratory infections; he was a homozygote for a frameshift variant.^8^

Recent research has been focused on the identification of a potential biomarker for NGLY1‐CDDG.^10^, ^12^ Hall et al demonstrated the usefulness of urine oligosaccharide profiling by MALDI‐TOF and the identification of a potential biomarker with a structure of Neu5Ac1Hex1GlcNAc1‐Asn (Asn‐N).^10^ Haijes et al reported aspartylglucosamine as the first small‐molecule biomarker identified in DBS by DI‐HRMS.^12^ Those biochemical methods are not routinely available yet and have still to be validated on a larger number of patients. Recently, Chang et al reported an additional NGLY1‐CDDG patient with typical clinical features with initially persistent, but resolving elevations in plasma methionine, plasma S‐adenosylmethionine (SAM), and plasma S‐adenosylhomocysteine (SAH), but with normal urine adenosine levels.^13^ Therefore, it is important to perform a careful clinical and biochemical examination due to the high specificity of selected signs and symptoms. As shown in Tables 2 and 3, global developmental disability was observed in all reported cases and it may hardly be considered to be highly specific for NGLY1‐CDDG. Hyperkinetic movement disorder as well as alacrimia/hypolacrimia were observed in most patients and are thus rather specific for NGLY1‐CDDG.

Elevated serum transaminases were reported in the majority of patients with normalization in some of them.^7^, ^9^ In our patient, serum transaminase elevation was first noted at 4 months of age. During a follow‐up at 21 months of age only a slight elevation of AST (<3 times ULN) accompanied with the normalization of alanine aminotransferase (ALT) was observed. A significant (>10 times the upper limit of normal range) elevation of both ALT and AST was noted during a period of increased catabolism (infection). To the best of our knowledge, this is the first report of such episode of liver involvement in an NGLY1‐CDDG patient.

In several NGLY1‐CDDG patients liver biopsy was performed showing cytoplasmic storage of amorphous material (with staining properties similar to glycogen) or vacuolization in hepatocytes consistent with storage.^6^, ^7^, ^8^, ^9^ Moreover, Heeley et al reported on a patient in whom liver biopsy performed at 6 months of age showed features of liver cirrhosis.^7^ In a patient diagnosed at 7 years of age with a urinary biomarker identified by Hall et al, liver biopsy done at 10 months of age was indicative of bridging fibrosis. One of the patients described by Lam et al underwent orthotopic liver transplantation at 21 months of age for liver cirrhosis and presumed hepatocellular carcinoma.^9^

**Figure 3:**
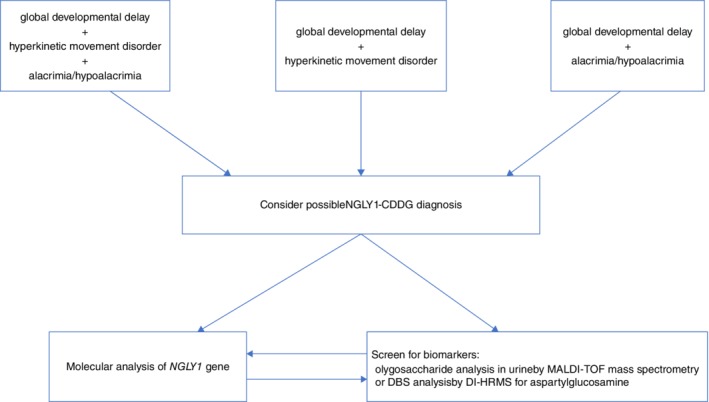
Diagnostic algorithm for NGLY1‐CDDG

Considering our results and the review of the literature we propose an algorithm for NGLY1‐CDDG diagnosis (Figure 3). NGLY1‐CDDG should be considered in patients with developmental disability associated with a hyperkinetic movement disorder and alacrimia/hypolacrima. Absence of the latter two symptoms does not rule out this diagnosis.

An increase of serum transaminases and of the above mentioned biomarkers are in favor of this diagnosis. In NGLY1‐CDDG patients, liver biochemistry tests and abdominal ultrasound should be performed. The evaluation of possible liver disease should not delay the diagnostics of NGLY1‐CDDG. We also propose that NGLY1‐CDDG should be considered in patients presenting with unexplained liver cirrhosis (Figure 3).

In patients suspected for NGLY1‐CDDG, the molecular analysis of the *NGLY1* gene should be performed. Two proposed diagnostic biomarkers for NGLY1‐CDDG are promising for the biochemical diagnosis.

In summary, this study reports an additional NGLY1‐CDDG patient, presents a review of the reported patients and proposes a diagnostic algorithm.

## CONFLICT OF INTEREST

The authors declare no potential conflict of interest.

## Supporting information


**Appendix S1.** Supporting Information
