# Supplementary material for: NGLY1 deficiency: Novel patient, review of the literature and diagnostic algorithm
Source: JIMD Rep. 2020 Jan 30;51(1):82–8. doi: 10.1002/jmd2.12086 (PMC7012742; doi:10.1002/jmd2.12086)
Supplement: Supplementary file 1 — Appendix S1. Supporting Information [file JMD2-51-82-s001.docx]

**Supplementary material**

*Assessment of serum lipid profile*

We determined parameters of lipid metabolism: total cholesterol (TC), total triglycerides (TG), high density lipoprotein – cholesterol (HDL-C), low density lipoprotein – cholesterol (LDL-C), very low density lipoprotein – cholesterol (VLDL-C) and apolipoproteins AI and B (ApoAI, ApoB). HDL-C and LDL-C lipoproteins were isolated by precipitation methods and the VLDL-C lipoproteins were isolated using the method of density gradient ultracentrifugation, and then in each of the isolated fractions cholesterol concentration was measured. ApoA1 and ApoB were determined by immunoturbitimetry using polyclonal goat. Lipoprotein a (Lpa) concentrations were determined by immunoelectrophoresis in agarose gels with specific antibodies.

*Anthrophological assessment*

The degree and direction of deviations of studied features in the patient were analysed using the data standardization method, and the calculated values were presented as z-scores, while the growth trend for body height, weight and head circumference was assessed using the straight-line regression model [16].

*Transient elastography*

Transient elastography by FibroScan® (Echosens, Paris, France) measurements were performed in the patient by the same medical doctor during routine visit in the Outpatient Clinic. Measurements were made before breakfast or at least 2 hours after a meal. The probe M (medium) was used and the liver stiffness (LS) was assessed. Quality criteria of transient elastography were the following: interquartile range <25 %, number of valid measurements – 10, LS reliability assessed as interquartile range to the median ≤25 %. Elevated LS was defined as greater than 6 kPa.

*Molecular study*

Molecular analysis was performed after obtaining informed consent from the patient’ parents. Venous blood samples were collected from the proband and his parents. Whole exome sequencing was performed in the proband DNA sample. The nomenclature of molecular variants follows the Human Genome Variation Society guidelines (HGVS, www.hgvs.org/mutnomen) according to NGLY1 reference sequence NM_018297.3, followed the Human Gene Mutation Database (HGMD Professional, https://portal.biobase-international.com/hgmd/) and ClinVar (<https://www.ncbi.nlm.nih.gov/clinvar/>).

**Reference**

[16] Palczewska I., Niedźwiecka Z. Somatic development indices in children and youth of Warsaw. Develop Period. Med. 2001; **5**(Suppl. 1):15-118.
